# The asymmetric impact of renewable energy consumption on the economic growth of emerging South and East Asian countries: A NARDL approach

**DOI:** 10.1016/j.heliyon.2023.e18656

**Published:** 2023-07-25

**Authors:** Sk Habibur Rahaman, Fuzhong Chen, Guohai Jiang

**Affiliations:** a School of International Trade and Economics, University of International Business and Economics, Beijing, 100029, China; b The Academy of China Open Economy Studies, School of International Trade and Economics, University of International Business and Economics, Beijing, 100029, China

**Keywords:** Renewable energy, Economic growth, Asymmetric impact, Causality

## Abstract

The question of economic development is so essential that specialists, international leaders, and every government are continually working on how to tackle this problem. Renewable energy is a means to save planet’s ecology and foster long-term economic viability. This study explores the asymmetric effect of renewable energy consumption (RE) on emerging South and East Asian countries’ economic growth by the non-linear autoregressive distributed lag (NARDL) approach. Also, it employs the generalized least square (GLS) method and panel causality test to grasp this impact. The GLS assessment exposes that positive and negative (P&S) mechanisms of RE positively influencing GDP while urbanization has an adverse influence. The PMG approach also delivers equivalent outcomes and authenticates the robustness of GLS results. The causality results provide relations between GDP and other variables. There is a conservation mechanism between the negative shock of RE consumption and GDP, while the positive shock of RE to GDP is observed from the feedback mechanism. We observed different interactions of CO_2_, P&S shocks of RE, and non-renewable consumption on GDP. These findings support policymakers of South and East Asian countries in formulating effective rules for their financial institutions regarding energy guidelines. In addition, considering P&S shocks from RE specifies that effective outcomes can be attained in economic growth while formulating energy guidelines.

## 1 Introduction

Consuming energy from non-renewable sources may be productive and promote economic growth, but there is little question that it also causes excessive carbon emissions and environmental damage [1]. Developing nations that rely on non-renewable energy sources must balance the competing goals of reducing pollution and expanding their economies. Therefore, energy should be utilized wisely and effectively since it has finite supplies. Furthermore, in light of the current global warming crisis, renewable energy (RE) may represent alluring alternative to tradition energy, allowing for decreased CO_2_ emissions. However, it is exceedingly time-consuming and expensive to introduce new renewable energy technologies, to have them consumed, and to make them accessible to the public. Conversely, nations often struggle to sustain their economies’ expansion and growth. Both emerging and industrialized countries need to balance economic development and climate change adaptation investment [2].

**Fig. 1:**
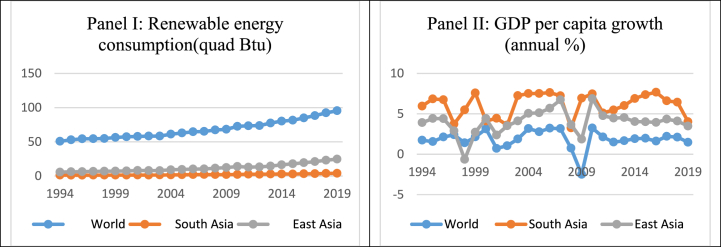
Trends of RE consumption and GDP growth.

According to Gabr & Mohamed [3] research, it is abundantly obvious that economic development is affected by energy consumption, which is a great source of greenhouse gases (GHG) emissions, especially carbon dioxide. Recently, countries have sought to structural shifts in industrial processes and energy consumption after suffering several catastrophes. In certain nations, renewable energy has been widely spread [4]. According to the Ernst & Young Company, three biggest economies, the America, China, and India, have been battling for this in the long run, offer the greatest opportunities for investments in renewables, which incorporates new global trends [5]. This company ranks the attractiveness of countries based on their potential for RE investment. Four hypotheses help to explain the energy consumption-economic growth nexus. They are known as different conclusions [[6], [7], [8], [9], [10], [11], [12], [13], [14]]. Some reveals a one-way positive relationship between them [[15], [16], [17], [18]]. Some reveals a two-way causality relationship, which argues economic growth would be reduced due to the conventional energy policy and would subsequently affect energy consumption negatively [[19], [20], [21], [22], [23]]. Also, some finds energy consumption doesn’t affect economic growth [[24], [25], [26]]. Furthermore, development indicators such as infant mortality, life expectancy, political and civil human rights, a clean environment, and GDP per capita are strongly correlated. However, Fig. 1 shows the relationship between the two is not strict.

**Fig. 2:**
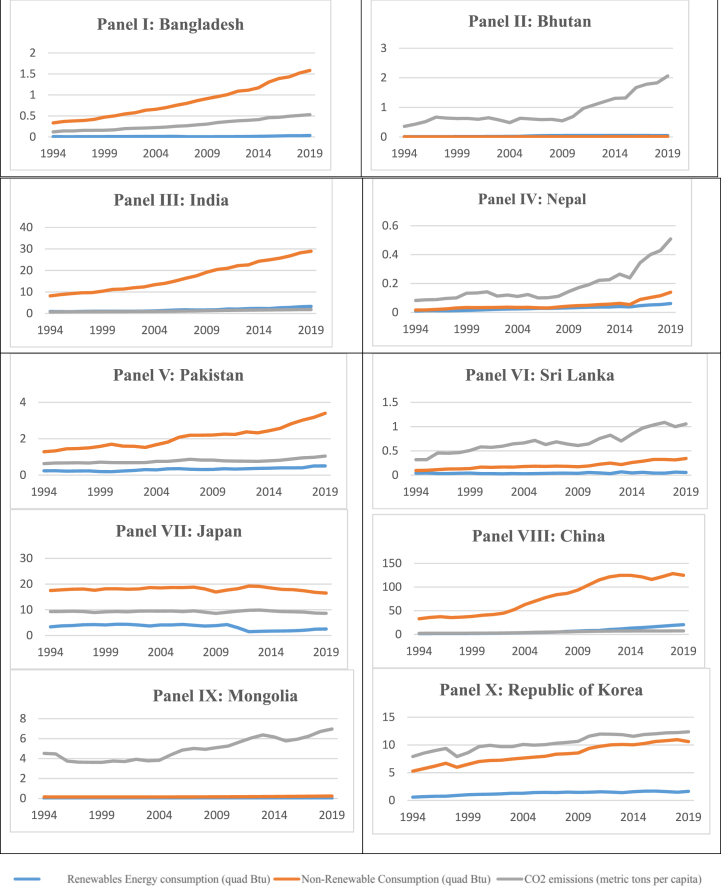
Trends of RE and carbon emissions in sample countries.

Fig. 2 summarize the baseline status of RE and carbon emissions in 10 representative countries.

The present research expands upon the aforementioned works in several ways: (i) it fills a major research gap on the South and East Asian economies; (ii) to prevent omitted variable bias, this study used a multivariate framework; (iii) control parameters were also included to further investigate the dynamic “asymmetric RE-GDP nexus”.

## 2 Literature review

### 2.1 Renewable energy consumption (REC) and economic growth

The research that was carried out by Li et al. [27] makes use of Chinese data between the years 2005 and 2017 to undertake an objective analysis of the non-linear impacts that REC have on economic growth and environment. This research shows that the correlation between REC and GDP expansion follows an N-curve. In Ghana, REC helps GDP growth [24]. In the BRICS economies, Ojekemi et al. [28] demonstrated that REC help to curb CO_2_ in from 1990 to 2018, while economic growth and imports were found to cause it [29].

However, the economic growth also hinders REC in Thailand and Turkey, according to the research of Eyuboglu & Uzar [30], who analyzed data from 15 developing countries between 1990 and 2015. The research that was done between the years 1990 and 2020 using the techniques provided by Hung [31].

An asymmetric positive relationship between REC and GDP was explored by Luqman and Ahmad [32]. Furthermore, Apergis and Payne [33,34] find the association within a multi-stakeholder framework for twenty OECD countries. A panel error correction and cointegration process are applied to conjecture the causal association for short-time series. There is long-term effects of REC on GDP. Both in the long and short-term, the bidirectional casual relation between REC and GDP is detected by the test of granger-causality [35,36]. However, many investigators examined this relationship for individual countries as well as for groups of countries, few of them are mentioned here coherently [37,38].

### 2.2 Carbon emissions, other variables, and economic growth

The relationships between carbon emissions and GDP are found in many countries, including ASEAN-6 countries [39], China [40], 124 countries [41], Turkey [42], France [43], South Asian countries [44,45], Upper Middle-Income Countries [46]. Khan et al. [47] demonstrated that reduced carbon emissions increase environmental quality. In a world where carbon emissions impacted the economic development of the world’s highest-income nations from 2002 to 2019, GDP is influenced by REC. Furthermore, as GHG emissions are known to slow economic development [48,49], it is important to make use of comparatively cleaner energy sources to optimize both economic and environmental prosperity. Cole [50] finds a significant prove of carbon emissions-income level nexus, which is identical to the EKC. To address this relationship of twenty-two organizations for OECD countries by well-designed form to cubic specification, from 1975 to 1998, according to the research of Martínez-Zarzoso & Bengochea-Morancho [51]. On the other hand, it makes sense that more fossil fuels must be used to boost a country’s GDP, resulting in more CO_2_ emissions. This logical assertion suggests that GDP and CO_2_ emissions may have a bidirectional connection. Due to the extensive range of data and study methodologies, previous findings are likely inconsistent.

The author is aware of relatively little literature that focuses on the asymmetric relationship between REC on economic development in South and East Asian countries. Therefore, this study’s purpose is to contribute in a variety of ways to the expansion of the existing body of literature: (a) It fills a major research gap on the South and East Asian economies. (b) It fills a large geographical knowledge gap by focusing on economies in both South and East Asia; (c) It’s the first study to use up-to-date information from both current South and East Asian regions; and (d) It’s the first to take into account industrial and population factors in its examination of REC-GDP nexus.

## 3 Methods

### 3.1 Sample and data source

This study employs 10 emerging South & East countries from 1994 to 2019 as sample. They are China, Mongolia, Republic of Korea, Bangladesh, Bhutan, India, Nepal, Pakistan, Sri Lanka, and Japan. Non-energy data might be found on the World Bank’s website. The information on energy use comes from EIA’s website and similar study is done by Adams et al. [52].

### 3.2 Model

The empirical model is an Aggregated Cobb-Douglas function used by Buhari, Lorente, and Ali Nasir; Le and Bao; and Shahbaz, Khan, and Tahir [[53], [54], [55]]. As a revision to Solow’s [56] growth model, the applied theoretical notion is generated as follows:

(1) $${GDP=f(NRE,RE,IND,PD,S,U,CO}_{2})$$

In Eq. (1), a model is formulated. GDP is determined by non-renewable (NRE) and renewable (RE), industrialization (IND), population density (PD), service sector (S) in GDP, urbanization (U), and CO_2_ emissions. Table 1 contains the data summaries.

**Table 1:** Descriptive statistics.

Var.	Obs	Mean	Std. dev.	Min	Max
LnGDP	260	12.72	2.526	7.326	16.807
LnNRE	260	0.049	2.914	−6.227	4.855
LnRE	260	−1.918	2.677	−7.983	3.022
LnIND	260	3.342	0.304	2.538	3.862
LnPD	260	4.958	1.81	0.384	7.133
LnS	260	3.872	0.195	3.378	4.275
LnU	260	3.607	0.571	2.345	4.519
CO2	260	3.359	3.772	0.083	12.368

In the panel dataset, cross-sectional dependency (CD) exists, detected by the CD test [57]. Given the misleading outcomes from the traditional test, we apply Pesaran [58] 2nd^,^ generation “Cross-sectional augmented Dickey-Fuller (CADF) unit root test” and Pesaran, Vanessa Smith, and Yamagata [59], CIPS unit root test. Here, the partial sum process, as outlined in Eq. (2.1) and Eq. (2.2) obtains the positive and negative shocks of REC. This research also helps us understand how nonlinearities and asymmetries affect short and long-term cointegration. However, in the previous research, the ARDL method was inadequate to inspect the nexus among the variables in short and long-run.

(2.1) $$RE\_POS_{t}=\sum_{i=t}^{t}\Delta RE\_POS_{i}=\sum_{i}^{t}\Delta \max\left(\Delta RE_{i,}0\right)$$

(2.2) $$RE\_NEG_{t}=\sum_{i=t}^{t}\Delta RE\_NEG_{i}=\sum_{i}^{t}\Delta \max\left(\Delta RE_{i,}0\right)$$

Here, RE^_POS^ and RE^_NEG^ symbolize REC’s positive and negative shocks. Equations (3), (4), (5) shows the panel least square regression of the Pooled Least Square, Fixed effects (FE), and Random effects (RE).

(3) $${GDP}_{it}=\alpha_{0}+\alpha_{1}{NRE}_{it}+\alpha_{2}{RE\_POS}_{it}+\alpha_{6}{RE\_NEG}_{it}+\alpha_{4}{IND}_{it}+\alpha_{5}{PD}_{it}+\alpha_{6}S_{it}+\alpha_{7}U_{it}+\alpha_{8}{{CO}_{2}}_{it}+\varepsilon_{it}$$

(4) $${GDP}_{it}=\alpha_{0}+\alpha_{1}{NRE}_{it}+\alpha_{2}{RE\_POS}_{it}+\alpha_{6}{RE\_NEG}_{it}+\alpha_{4}{IND}_{it}+\alpha_{5}{PD}_{it}+\alpha_{6}S_{it}+\alpha_{7}U_{it}+\alpha_{8}{{CO}_{2}}_{it}+\varepsilon_{it}$$

(5) $${GDP}_{it}=\alpha_{0}+\alpha_{1}{NRE}_{it}+\alpha_{2}{RE\_POS}_{it}+\alpha_{6}{RE\_NEG}_{it}+\alpha_{4}{IND}_{it}+\alpha_{5}{PD}_{it}+\alpha_{6}S_{it}+\alpha_{7}U_{it}+\alpha_{8}{{CO}_{2}}_{it}+\varepsilon_{it}+\varepsilon_{it}$$

There is heteroskedasticity in the panel dataset, which is detected by the heteroskedasticity LR test. A GLS model is applied for this panel, which is superior and more efficient than the panel LS methods. We divided both sides of Eq. (3) and rearranged the output in Equation (6):

(6) $$\frac{{GDP}_{it}}{\sigma_{i}}=\frac{\alpha_{0}}{\sigma_{i}}+\frac{\alpha_{1}{NRE}_{it}}{\sigma_{i}}+\frac{\alpha_{2}{RE\_POS}_{it}}{\sigma_{i}}+\frac{\alpha_{3}{RE\_NEG}_{it}}{\sigma_{i}}+\frac{\alpha_{4}{IND}_{it}}{\sigma_{i}}+\frac{\alpha_{5}{PD}_{it}}{\sigma_{i}}+\frac{\alpha_{6}S_{it}}{\sigma_{i}}+\frac{\alpha_{6}U_{it}}{\sigma_{i}}+\frac{\alpha_{7}{{CO}_{2}}_{it}}{\sigma_{i}}+\frac{\varepsilon_{it}}{\sigma_{i}}$$

The rearranged equation is rewritten in the simple form, Eq. (7), where homoscedasticity and inefficiency remain. From Eq. (7), we examine the nexus among the variables. To validate the appropriateness of the GLS model, a set of diagnostic tests are applied. Furthermore, Pesaran et al. (2009) check the GLS outcome’s robustness.

(7) $${GDP}_{it}^{*}=\alpha_{0}+\alpha_{1}{NRE}_{it}^{*}+\alpha_{2}{RE\_POS}_{it}^{*}+\alpha_{3}{RE\_NEG}_{it}^{*}{+\alpha }_{4}{IND}_{it}^{*}+\alpha_{5}{PD}_{it}^{*}+\alpha_{6}S_{it}^{*}+\alpha_{7}U_{it}^{*}+\alpha_{8}{{CO}_{2}^{*}}_{it}+\mu_{it}$$

In Equation (8), the PMG model considers error variance and short and long-run cointegrations.

(8) $$\Delta {GDP}_{it}=\sum_{j=1}^{p-1}\vartheta_{ij}\Delta {GDP}_{it-j}+\sum_{j=0}^{q-1}\theta_{ij}\Delta X_{it-j}+\delta_{i}\left[{GDP}_{it-1}-\beta_{i}X_{it-1}\right]+\mu_{i}+\varepsilon_{it}$$

here, i symbolizes the quantity of cross-sections, t displays time, j narrates to time lag, Vector of explanatory variables is $X_{i}$, and $\delta_{i}$ indicates the speed of adjustment of GDP. If ***δ***_i_ < 0 and a significant value indicates cointegration and long-run association among the variables. $\mu_{i}$ indicates fixed effect.

The direction among the variables and the existence of causality is determined by Equation (9).

(9) $$Y_{it}=\beta_{i}+\sum_{i=1}^{k}\alpha_{i}Y_{i,t-k}+\sum_{i=1}^{k}\delta_{i}X_{i,t-k}+\varepsilon_{i,t}$$

where ***δ***_i_, ***α***_I,_ and ***β***_1_ indicate the coefficient slope, lag, and constant, respectively. ***δ***_I_ determines whether the causality exists.

## 4 Results

### 4.1 Outcomes of CD and URT test

Table 2 represents the cross-sectional dependency (CD) test results [56], indicating variables are dependent or correlated.

**Table 2:** Cross-section dependence test.

Test	LnGDP	LnNRE	LnRE	LnIND	LnPD	LnS	LnU	CO2
Breusch-Pagan LM	1049.23***	840.50***	632.82***	277.86***	954.80***	734.54***	903.37***	738.27***
Pesaran scaled LM	105.85***	83.85***	61.96***	24.54***	95.90***	72.68***	90.48***	73.07***
Bias-corrected scaled LM	105.65***	83.65***	61.76***	24.34***	95.70***	72.48***	90.28***	72.87***
Pesaran CD	32.31***	24.33***	15.73***	0.67	30.09***	26.64***	23.33***	22.47***

Table 2 reveals that Cross-sectional dependency exists in the panel dataset. They indicates the variables are I(1) process. This applies panel NARDL method.

### 4.2 Cointegration test

We analyze the connections between the variables using a Kao panel cointegration method. This method also permits cross-sectional independence, which is useful for figuring out whether or not a long-term or cointegration link really exists. The results indicates that Augmented Dickey-Fuller statistics is −4.815 and p-value < 0.001, which corroborates the cointegration relationships.

### 4.3 Results of panel least square

In Table 3, FE outcomes are examined for “the redundant fixed effects test” which is significantly rejected (Chi-square = 484.99 and p-value < 0.001).

**Table 3:** Cross-sectional FE outcomes.

Var.	Coef.	Std. Err.	t	P
LnNRE	0.332	0.054	6.155	0.000
LnRE^_NEG^	−0.057	0.025	−2.258	0.024
LnRE^_POS^	0.168	0.025	6.662	0.000
LnIND	0.731	0.100	7.284	0.000
LnPD	1.346	0.165	8.122	0.000
LnS	0.834	0.160	5.202	0.000
LnU	−0.188	0.128	−1.471	0.142
CO2	0.072	0.013	5.493	0.000
C	0.586	0.998	0.587	0.557

Also, Table 4 shows RE outcomes. The Hausman test shows that Chi-square = 58.248 and p < 0.001.

**Table 4:** Cross-sectional RE outcomes.

Var.	Coef.	Std. Err.	t	P
LnNRE	0.591	0.035	16.449	0.000
LnRE^_NEG^	−0.029	0.024	−1.189	0.235
LnRE^_POS^	0.194	0.020	9.524	0.000
LnIND	0.923	0.092	9.958	0.000
LnPD	0.485	0.061	7.892	0.000
LnS	1.086	0.152	7.101	0.000
LnU	−0.431	0.111	−3.886	0.000
CO2	0.041	0.012	3.579	0.000
C	4.191	0.803	5.220	0.0000

Therefore, we run Panel LS, and its outcomes suggest heteroskedasticity, which is indicated in Table 5.

**Table 5:** Panel PLS outcomes.

Var.	Coeff.	Std. Err.	t	P
LnNRE	0.780	0.010	75.322	0.000
LnRE^_NEG^	0.290	0.048	6.040	0.000
LnRE^_POS^	0.168	0.028	6.016	0.000
LnIND	0.563	0.08	7.032	0.000
LnPD	0.201	0.02	9.736	0.000
LnS	1.658	0.195	8.489	0.000
LnU	−0.671	0.087	−7.748	0.000
CO2	0.004	0.012	0.354	0.723
C	5.747	0.846	6.795	0.000

The Panel GLS model is applied based on “cross-sectional seemingly unrelated regressions” in these circumstances. The results of the redundancy test, correlation-Hausman test, and cross-sectional fixed and random effects tests conducted using panel GLS.

Table 6 shows the panel pooled GLS estimation for the inappropriateness of fixed and random effects tests. In addition to confirming the normality of error terms and the absence of CD, our tests also found that panel GLS findings are acceptable. Furthermore, the results are free from heteroskedasticity at a 1% significance level and efficiency. The coefficients of RE^−POS^ and RE^−NEG^ reveals long-run positive effects on GDP exist among them. Accordingly, RE consumption’s positive and negative shocks swell economic growth.

**Table 6:** GLS outcomes.

Var.	Coef.	Std. Er.	t	P
LnNRE	0.294	0.038	7.631	0.000
LnRE^_NEG^	0.018	0.009	2.000	0.046
LnRE^_POS^	0.163	0.017	9.276	0.000
LnIND	0.383	0.060	6.323	0.000
LnPD	1.176	0.054	21.72	0.000
LnS	0.430	0.123	3.474	0.000
LnU	0.301	0.101	2.969	0.003
CO2	0.048	0.007	6.385	0.000
C	2.509	0.658	3.812	0.000

Non REC sources have been shown the trend that is in concordance with a country’s economic prosperity. Table 6 reveals that the corresponding elasticity is 0.2946. These results align with the previous findings [60,61], which demonstrate that NRE consumption contributes to economic development. In contrast, the study's findings by Mohamed et al. [62], which finds that NRE consumption hinders economic growth. The economy grows by 0.0185% points at the 1% significance level for every one percent increase in negative shock of renewable energy use. Both model (PMG and GLS) findings of RE consumption correspond to the outputs of Rafindadi & Ozturk, and Atems & Hotaling [63,64] as well as contrast with the result of Venkatraja and Tsaurai & Ngcobo [65,66] because of higher adaptation costs and lack of enlightening access respectively but their magnitude are different. A positive change in renewable energy contributes 0.1634% points to economic growth. Consistent with the results of Rafindadi & Ozturk, and Atems & Hotaling [63,64] and in contrast to the results of Venkatraja and Tsaurai & Ngcobo [65,66] due to higher transferring costs and a lack of instructive access, both models (PMG and GLS) find that people are increasingly using renewable energy. The long-term elasticity of industrialization, population density, the service sector in GDP, and urbanization are positive and significant at 1%. They are 0.383, 1.176, 0.430, and 0.301, respectively. Carbon emissions also positively correlate with economic development and the elasticity is 0.048, which is consistent with previous research [[67], [68], [69], [70]].

Some studiesargued that using renewable energy sources contributed to a flourishing economy, such as [71,72]. Long-term asymmetry testing results also confirmed that RE consumption’s positive and negative shocks produced a long-run asymmetric impact. Therefore, the asymmetric effects of LnRE_POS and LnRE_NEG shocks on economic expansion set up.

### 4.4 Pooled mean group (PMG) outcomes

A non-linear ARDL method-based PMG model is used, which is defined as NARDL (2, 1, 1, 1, 1, 1, 1, 1, 1). The automatic selection max dependent lags are 2. The dynamic repressor is 1, which is automatically selected following the AIC. Table 7 presents the NARDL (2, 1, 1, 1, 1, 1, 1, 1, 1) outcomes. The coefficient of NRE is significant. The results of PMG model estimation confirm the results of panel GLS. The long-run GLS coefficients are significantly positive, which confirms that the asymmetric shocks of REC positively affect GDP; these results are robust to the GLS outcomes. GDP is boosted by 0.04 due to the negative shock of RE at the same time, 0.1319 increasing due to the positive shock of RE consumption. The coefficients of RE^_POS^ and RE^_NEG^ are positive, but their magnitudes are different, which confirms that they are significant to economic growth. This output of PMG outcomes is robust to the panel GLS results. The coefficients of industrialization (IND) are significantly positive at 1% levels, which mean that GDP is growing by 38.69% due to the 1% increase in industrialization (IND). These outcomes align with Ndiaya et al. [73].

**Table 7:** Pooled mean group outcomes.

Var.	Coeff.	Std. Err.	t	P
	Long Term Equ.			
LnNRE	0.351	0.068	5.096	0.000
LnRE^_NEG^	0.131	0.013	9.807	0.000
LnRE^_POS^	0.040	0.007	5.616	0.000
LnIND	0.386	0.046	8.376	0.000
LnPD	0.904	0.218	4.142	0.000
LnS	0.228	0.074	3.064	0.002
LnU	0.851	0.080	10.60	0.000
CO2	0.028	0.013	2.198	0.029
	Short Term Equ.			
COINTEQ01	−0.502	0.156	−3.216	0.001
D (LnGDP (−1))	0.240	0.113	2.12	0.035
D (LnNRE)	−0.196	0.115	−1.695	0.092
D (LnRE^_POS^)	−0.076	0.052	−1.447	0.150
D (LnRE^_NEG^)	−0.087	0.121	−0.716	0.475
D (LnIND)	−0.298	0.215	−1.381	0.169
D (LnPD)	−4.300	8.856	−0.485	0.628
D (LnS)	−0.743	0.379	−1.958	0.052
D (LnU)	1.154	2.311	0.499	0.618
D (CO2)	0.121	0.125	0.971	0.333
C	1.710	0.643	2.658	0.008

The coefficients of population density (PD) are positive and significant at 1% levels, demonstrating a long-run positive influence on economic growth, implying that GDP is expanding by 90.44 % due to the 1% increase in PD. Service sector’s coefficients are significantly positive, confirming that the service sector (S) helps economic growth. This result means that GDP grows by 22.82% as the service sector (S) GDP increases by 1%. To prove the long-term beneficial influence on economic growth, the urbanization coefficients (U) are positive and significant at 1% levels, increasing GDP by 85.13% due to the 1% rise in urbanization (U). Overall, we can claim that the long-term coefficients are all significantly positive at 5% levels. Due to a 1% rise in them, GDP is expanding by 38.69%, 90.44%, 22.82%, and 85.13%, respectively. A one-percentage-point rise in CO2 emissions helps GDP to improve 2.87%, according to the coefficients of CO2 emissions. It is found that CO2 emissions are helpful to GDP growth in the America, Japan, and China but this finding is comparable [74]. The long-run coefficient of NRE consumption and “RE_POS and RE_NEG” are negative and insignificant at the 5% level. It also reveals that the system is approaching long-term equilibrium at 50.26% per year. All variables’ coefficients are affirmative and significant, meaning that these variables have a long-term positive impact on GDP, which is consistent with previous research [[68], [69], [70]].

### 4.5 Causality test

Table 8, Appendix I (Fiigure 3) further summarizes this study's causal relationships. The six bidirectional causality and feedback mechanisms happen with GDP, between NRE↔GDP; PD↔GDP; S↔GDP; U↔GDP; RE^_POS^↔GDP; CO2↔GDP. At the same time, two unidirectional causality and conservation mechanisms happen between GDP→IND; GDP→ RE^_NEG^. These causalities validate that the outcomes are not accepted at a 1% significance level. Overall, twenty bidirectional, thirteen unidirectional, and two no-cause causalities were found in the D-H causality test findings, shown in Appendix I (Fiigure 3). The twenty-bidirectional causality is NRE↔GDP; PD↔GDP; S↔GDP; U↔GDP; RE^_POS^↔GDP; CO2↔GDP; PD↔NRE; U↔NRE; PD↔IND; U↔IND; S↔PD; U↔PD; RE^_NEG^↔PD; RE^_POS^↔PD; CO2↔PD; U↔S; RE^_POS^↔S; RE^_NEG^↔U; RE^_POS^↔ RE^_NEG^; CO2↔ RE^_NEG^. The first bidirectional causality relation between NRE and GDP means that the more NRE, the more industrialization and GDP, and vice versa. Second, a bidirectional causality between population density and GDP is detected, implying that countries with strong economies prioritize investing in their citizens' education and training to serve their citizens better and contribute to the expansion of their economies. The third two-way causality association between services in GDP and economic growth suggests that nations with more robust economies focus on providing better services to their citizens, which favours economic growth. Fourth, a significant relationship between urbanization and GDP is found in nations of varying levels of development. An answered issue from this fifth two-way causality is that economic development promotes urbanization, and urbanization promotes economic growth.

**Table 8:** Results of panel causality.

Relationship	W-stat.	Zbar-Stat.	P	Conclusion
LnNRE → LnGDP	4.608	2.982	0.002	LnNRE ↔LnGDP bidirectional
LnGDP → LnNRE	6.838	5.786	0.000	
LnIND → LnGDP	3.280	1.314	0.188	LnGDP → LnIND unidirectional
LnGDP → LnIND	5.604	4.235	0.000	
LnPD → LnGDP	8.441	7.800	0.000	LnPD ↔LnGDP bidirectional
LnGDP → LnPD	12.116	12.42	0.000	
LnS → LnGDP	6.835	5.782	0.000	LnS ↔LnGDP bidirectional
LnGDP → LnS	5.312	3.868	0.000	
LnU → LnGDP	8.806	8.260	0.000	LnU ↔LnGDP bidirectional
LnGDP → LnU	3.793	1.958	0.050	
LnRE^_NEG^→ LnGDP	3.748	1.857	0.063	LnGDP → LnRE_NEG unidirectional
LnGDP → LnRE^_NEG^	7.512	6.523	0.000	
LnRE^_POS^→ LnGDP	6.567	5.350	0.000	LnRE_POS ↔LnGDP bidirectional
LnGDP → LnRE^_POS^	6.368	5.104	0.000	
CO2 → LnGDP	5.265	3.809	0.000	CO2 ↔LnGDP bidirectional
LnGD → CO2	8.039	7.296	0.000	

## 5 Conclusions and implications

This paper evaluates the asymmetric influence of the use of renewable energy (RE) on economic development in selected rising nations in South and East Asia between the years 1994 and 2019. The results of the GLS analysis suggest that the economy's expansion results from a number of diverse forces working together. These elements include of non RE sources, the upgrade of industries, the concentration of populations, and carbon emissions. According to the results, the asymmetric effect of RE sources improves GDP of South and East Asia. However, using NRE sources has a considerably substantial influence on GDP than using renewable energy or other environmentally friendly sources. According to the findings of PMG, the expected long-term elasticity of energy from green sources (renewable energy) is between 0.131 and 0.040, whereas the elasticity of conventional energy sources is 0.351. Also, the causality between carbon emissions and GDP is two-way, with both factors having an impact on the other.

We discover six “two-way” and two “one-way” links between the variables and GDP. The six bidirectional causality and feedback mechanisms happen with GDP, between NRE↔GDP; PD↔GDP; S↔GDP; U↔GDP; RE^_POS^↔GDP; CO2↔GDP. At the same time, two unidirectional causality and conservation mechanisms happen between GDP→IND; GDP→ RE^_NEG^. These causalities validate that the outcomes are not accepted at a 1% significance level. Overall, twenty bidirectional, thirteen unidirectional, and two no-cause causalities were found in the D-H causality test findings, shown Appendix I (Fiigure 3). The twenty-bidirectional causality is NRE↔GDP; PD↔GDP; S↔GDP; U↔GDP; RE^_POS^↔GDP; CO2↔GDP; PD↔NRE; U↔NRE; PD↔IND; U↔IND; S↔PD; U↔PD; RE^_NEG^↔PD; RE^_POS^↔PD; CO2↔PD; U↔S; RE^_POS^↔S; RE^_NEG^↔U; RE^_POS^↔ RE^_NEG^; CO2↔ RE^_NEG^. The significance threshold for this finding is a 5% level. The positive increase in RE sources, the consumption of NRE sources, services supply, the population density, urbanization, and carbon emissions are factors that helps to the expansion of the economy in a manner that is bidirectional. However, there is only one direction of causation between GDP and industrialization and the negative shock caused by the usage of renewable energy.

It would seem that different energy uses have different effects on GDP, and disaggregated energy consumption with both positive and negative shocks of RE confirms that these effects are positive on GDP. The greater the number of applications for renewable energy, the higher the GDP, and the better the environmental quality, the more impressive these conclusions are. Already, this demonstrates that the utilization of RE sources is kind to the environment. When it comes to their attempts to lessen environmental pollution, the people in charge of making decisions in the nations that were investigated would see this as a much-appreciated reprieve. As a consequence, the study's findings may inspire the establishment of legislation to enhance sustainable energy.

This research has some limitations. The study panel is confined to just 10 rising South and East Asian nations, and it might be expanded to analyze economic growth performance concerning energy and emissions. In this study’s sample, adding other external factors such as trade openness, labor, and capital is feasible. In light of the EKC mechanism, it is possible that further study will use the same panel to explore the asymmetric influence that disaggregated energy usage has on economic development and carbon emissions.

## Author contribution statement

Sk Habibur Rahaman: Performed the experiments; Analyzed and interpreted the data; Contributed reagents, materials, analysis tools or data; Wrote the paper. Fuzhong Chen: Contributed reagents, materials, analysis tools or data; Wrote the paper. Guohai Jiang: Conceived and designed the experiments; Contributed reagents, materials, analysis tools or data; Wrote the paper.

## Data availability statement

Data associated with this study has been deposited at https://databank.worldbank.org/source/world-development-indicators, an open-source online data repository hosted at The World Bank.

https://www.eia.gov/international/data/world#/?, an open-source online data repository hosted at U.S Energy Information Administration.

## Declaration of competing interest

The authors declare that they have no known competing financial interests or personal relationships that could have appeared to influence the work reported in this paper.

## Figures and Tables

**Fig. 3:**
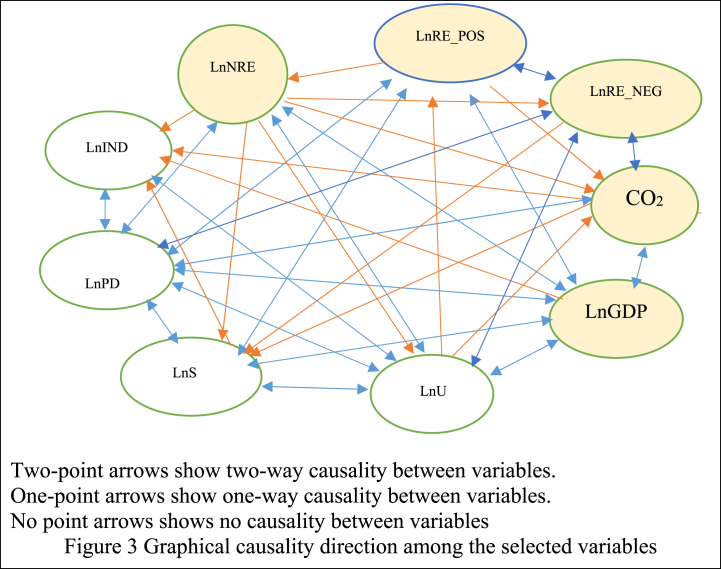
Graphical causality direction among the selected variables. Two-point arrows show two-way causality between variables. One-point arrows show one-way causality between variables. No point arrows shows no causality between variables.
